# Screening and identification of the H1R antagonists from natural products by BODIPY FL histamine recognition and DPHD-anchored bombardment coupled with target cell extraction

**DOI:** 10.3389/fphar.2025.1601384

**Published:** 2025-05-30

**Authors:** Xinqi Li, Guizhou Hu, Xu Chen, Can Di, Jin Qi

**Affiliations:** ^1^ Research Center for Traceability and Standardization of TCMs, School of Traditional Chinese Pharmacy, China Pharmaceutical University, Nanjing, China; ^2^ State Key Laboratory on Technologies for Chinese Medicine Pharmaceutical Process Control and Intelligent Manufacture, Jiangsu Kanion Pharmaceutical Co., Ltd., Lianyungang, China; ^3^ BYHEALTH Institute of Nutrition and Health, Guangzhou, China

**Keywords:** BODIPY FL histamine, H1R antagonists, target cell extraction, HPLC-Q-TOF-MS, natural products, dictamnine, pseudoephedrine

## Abstract

**Introduction:**

Histamine is an important mediator of allergy, and inhibiting its binding to H1 receptors (H1R) is a key method to alleviate allergic diseases. Natural products with anti-allergic properties are an important source of natural H1R antagonists.

**Methods:**

In this study, a rapid method for identifying the H1R antagonists from natural products via the BODIPY FL histamine recognition and diphenhydramine (DPHD)-anchored bombardment coupled with target cell extraction was developed. In addition, the activity of the H1R antagonist was further validated both *in vitro* and *in vivo* through BODIPY FL histamine recognition, intracellular fluorescence calcium ion (Ca^2+^) kinetic recognition, molecular docking, and animal experiments.

**Results:**

The binding of fluorescent histamine to H1R was notably inhibited by *Ephedra sinica* Stapf (ESS) and *Dictamnus dasycarpus* Turcz (DdT). Ephedrine and pseudoephedrine in ESS and dictamnine and limonin in DdT were screened as potential H1R antagonists using the target cell extraction of the DPHD-anchored bombardment. The BODIPY FL histamine recognition results revealed the significant blocking effects on H1R binding by pseudoephedrine (50 μM) and dictamnine (100 μM). Pseudoephedrine (200 μM) and dictamnine (100 μM) markedly decreased the histamine-induced increase in intracellular calcium ion (Ca^2+^) concentration. Docking results indicated strong binding affinity for both components to H1R, with dictamnine exhibiting a higher affinity than pseudoephedrine. Ultimately, the ameliorative effect of dictamnine on allergic rhinitis mice was confirmed through nasal symptom score, serum pharmacodynamic indices (immunoglobulin E (IgE), histamine, IL-2, IL-4, IL-6, and TNF-α), and histopathology.

**Conclusion:**

This study showed that dictamnine (validated *in vitro* and *in vivo*) and pseudoephedrine (validated *in vitro*) may serve as potential H1R antagonists. This study offered valuable insights for future developments in antihistamines.

## 1 Introduction

According to the 2020 White Paper published by the World Allergy Organization, allergic diseases affect 40% of the global population, encompassing 400 million cases of allergic rhinitis (AR), urticaria, and anaphylaxis (Cardona et al., 2020; Zhang et al., 2021). Histamine, which plays a crucial role in the development of allergy diseases, is released by mast cells upon allergen stimulation. It subsequently binds to histamine receptors, thereby initiating a cascade of allergic reactions (MacGlashan, 2003; Voelker and Pongdee, 2025). Histamine receptors are classified as G-protein-coupled receptors (GPCRs) and comprise four subtypes: histamine H1 receptors (H1R), H2 receptors (H2R), H3 receptors (H3R), and H4 receptors (H4R) (Tiligada et al., 2024). H1R are predominantly found on endothelial cells, and they regulate vasodilation and serve as the primary receptors in allergic diseases (Strasser et al., 2013). The interaction between histamine and H1R could induce plasma extravasation and vasodilation, ultimately resulting in allergy (Panesar et al., 2013). Consequently, inhibiting the combination of histamine and H1R can be an important approach to treat allergic diseases. Currently, the main antihistamines are H1R antagonists such as loratadine, diphenhydramine (DPHD), and ketotifen (Simons and Simons, 1994), but they often cause sedation and drowsiness. In addition, natural products exhibiting anti-allergic properties constitute a significant source for natural H1R antagonists (Xiao et al., 2020; Abd Rani et al., 2021; Um et al., 2021).

Fluorescent ligands combine known ligands with fluorophores to locate receptors. High-resolution GPCR structures enhance fluorescent ligand selectivity (Stoddart et al., 2018). Anni et al. employed a recognized dopamine receptor antagonist conjugated with Cy3B fluorescein to elucidate the binding characteristics between the ligand and receptor (Allikalt et al., 2021; Tahk et al., 2023). These demonstrated that GPCR fluorescent ligands exhibited specificity, high sensitivity, and traceability, thereby serving as a pivotal method in drug discovery (Arruda et al., 2017). Human umbilical vein endothelial cells (HUVECs) exhibited high expression of H1R, which is closely related to allergic diseases (Cao et al., 2020). By integrating fluorescent ligand technology, the antagonistic effects of natural products on H1R can be elucidated, yet isolating individual components that exhibit antagonism toward H1R from complex natural product systems remains challenging.

Target cell extraction has played an increasingly important role in the search for bioactive compounds in natural products (Hu et al., 2021; Huang et al., 2023; Chen et al., 2024), which selects the membrane receptor-associated ligands by utilizing living cells and mitigates the risk of false positives associated with purified receptor screening (Soave et al., 2020). Although HUVECs expressed H1R at high levels, other interfering receptors were also present (Adderley et al., 2015). Despite its widespread use in screening Chinese medicinal ingredients, target cell extraction does not facilitate the targeting of a specific receptor. DPHD, as a first-generation antihistamine with strong binding ability to H1R, was used clinically for the treatment of AR and urticaria (Wang et al., 2024). To accurately screen for components in natural products that bind to H1R on HUVECs, the concept of “anchored bombardment” with DPHD was introduced. Specifically, traditional Chinese medicine was incubated with the cells, followed by the addition of DPHD. DPHD anchored to the H1R binding site and displaced the components of the traditional Chinese medicine already bound to H1R through leveraging its strong binding affinity for H1R. By comparing the herbal components between groups with and without the addition of DPHD, the H1R-bound components of the natural products were identified.

**FIGURE 1:**
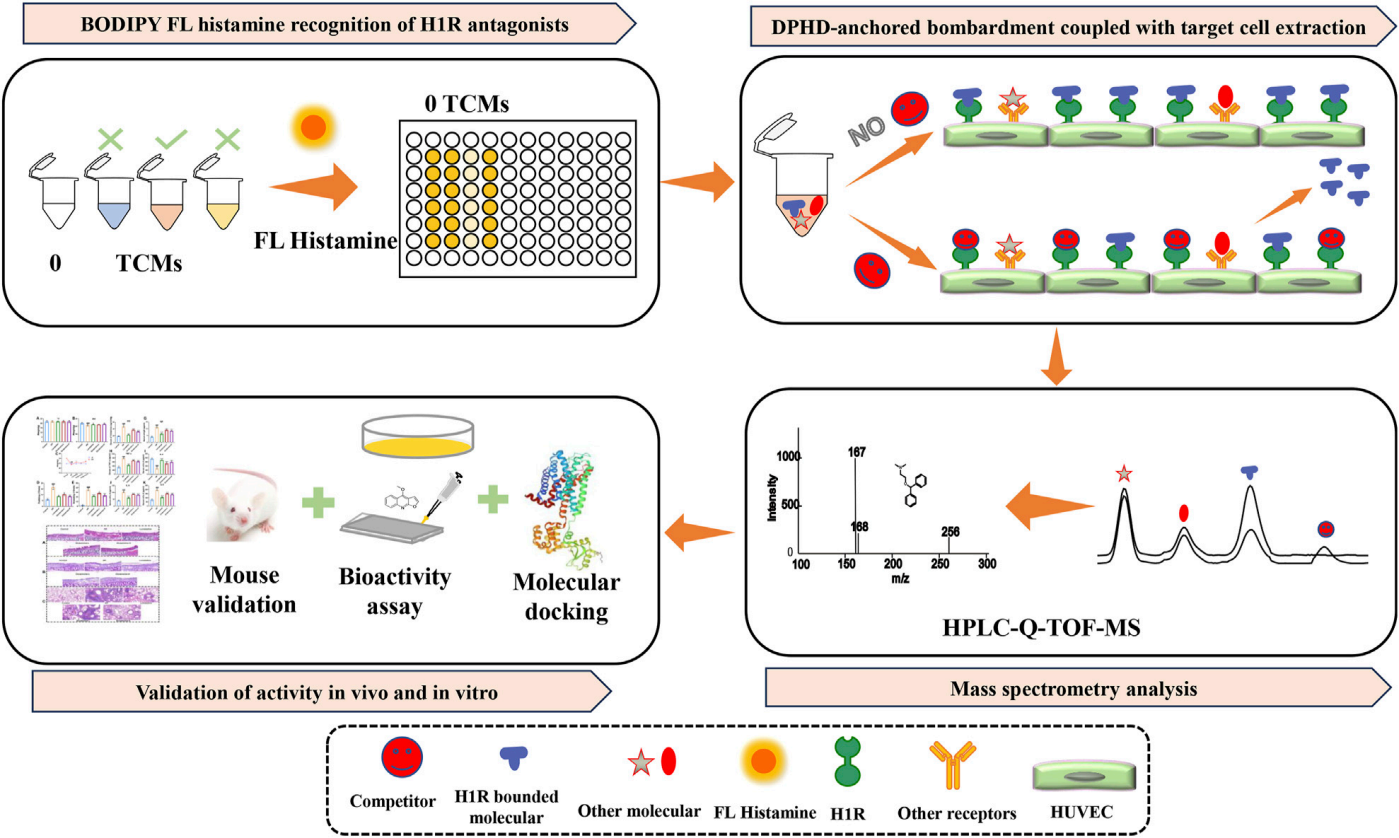
Rapid method of identifying the H1R antagonists using the BODIPY FL histamine recognition and DPHD-anchored bombardment coupled with target cell extraction.

In conclusion, this study employed HUVECs as the research subject, and a rapid and accurate method was constructed to identify H1R antagonists from natural products using BODIPY FL histamine recognition and DPHD-anchored bombardment coupled with target cell extraction (Figure 1). In addition, the activity of the H1R antagonist was further validated both *in vitro* and *in vivo* through BODIPY FL histamine recognition, intracellular fluorescence calcium ion (Ca^2+^) kinetic recognition, molecular docking, and animal experiments. This study provides a reference for identifying the effective and safe H1R antihistamines and GPCR antagonists.

## 2 Materials and methods

### 2.1 Reagents and materials

BODIPY FL histamine was purchased from Chutai Biotechnology Co., Ltd. (Shanghai, China). DPHD, histamine, ovalbumin, aluminum hydroxide (Al (OH)_3_), 1640 medium, and loratadine were purchased from Sigma-Aldrich (Saint Louis, United States). Fluo-4 AM was bought from Beyotime Co. Ltd. (Shanghai, China). Dimethyl sulfoxide (DMSO) was purchased from Titan Co., Ltd. (Shanghai, China). The Milli-Q ultrapure water meter was supplied by Millipore (Bedford, MA, United States). HPLC-grade acetonitrile was purchased from Tedia (Fairfield, OH, United States). HPLC-grade methanol was purchased from Nanjing chemical reagent Factory (Nanjing, China). Fetal bovine serum (FBS) was purchased from ExCell Bio Co., Ltd. (Suzhou, China). Streptomycin and penicillin were supplied by Amresco (Framingham, United States). HUVECs were purchased from Shanghai Cell Bank, Chinese Academy of Sciences (Shanghai, China). Fluorescent enzyme labeler was obtained from Thermo Fisher Scientific Co. Ltd. (Massachusetts, America).

*Ephedra sinica* Stapf (ESS) and *Dictamnus dasycarpus* Turcz (DdT) were offered by the Nanjing Traditional Chinese Medicine Clinic (Nanjing, Jiangsu, China) and were also appraised by Prof. Qi Jin of China Pharmaceutical University from the Traditional Chinese Medicine at China Pharmaceutical University. Ephedrine (purity ≥98%) and pseudoephedrine (purity ≥98%) were obtained from National Drug Reference Standards (Beijing, China). Dictamnine (purity ≥98%) and limonin (purity ≥98%) were obtained from Chengdu Efa Biotechnology Co., Ltd. (Chengdu, China).

### 2.2 Sample preparation of natural products

DdT: 1 g of the powder of DdT was weighed and taken in a conical flask after passing through a 40-mesh sieve, 20 mL of 95% (V/V) ethanol was added, and the extract was ultrasonicated at 100 Hz (30 min). The extraction was repeated once, and the two filtrates were combined and concentrated. The extract was dissolved by DMSO to obtain the reserve solution of the DdT extract, which was stored at 4°C.

ESS: 1 g of the powder of ESS was weighed and taken in a conical flask after passing through a 40-mesh sieve, 10 times the amount of 85% (V/V) ethanol was added, and the extract was ultrasonicated at 100 (30 min). The combined filtrates underwent concentration. The extract was dissolved by DMSO to obtain the reserve solution of the ESS extract, which was stored at 4°C.

### 2.3 Feasibility and optimization of the BODIPY FL histamine recognition of H1R antagonists

After 0.8 × 10^4^ cells per well were spread in 96-well plates and cultivated for 24 h, the cells were cultivated for 2 h with DPHD (1.25, 2.5, 5, 10, and 20 μM) and loratadine (1.25, 2.5, 5, 10, and 20 μM); then, BODIPY FL histamine (1 μM) was added. Only 1 μM of the BODIPY FL histamine solution was added to the control group. After washing six times with PBS, the fluorescence intensity was measured at an excitation wavelength of 488 nm and an emission wavelength of 520 nm.

After 0.8 × 10^4^ cells per well were spread in 96-well plates and incubated for 24 h, it was followed by the addition of DPHD (20 μM) for 0.5, 2, 4, and 6 h. Subsequently, the BODIPY FL histamine was cultivated with different concentrations (0.25, 0.5, 1, and 2 μM) for 15, 30, 45, and 60 min, with four replicate wells in each group. Subsequently, the cells were washed with PBS, the washing solution was transferred to a new 96-well plate, and the fluorescence intensity value was detected to examine the number of washes. The washed cells were detected at excitation (488 nm) and emission (520 nm) wavelengths. The results were expressed as the inhibition rate and repeated three times. The inhibition rate of the BODIPY FL histamine binding to H1R antagonist was calculated as follows:

$$\text{Inhibition rate }\left(\%\right)=\left(1-{\mathrm{FI}}_{\mathrm{dosing}}/{\mathrm{FI}}_{\mathrm{blank}}\right)\times 100\%.$$

Here, FI _blank_ is the fluorescence intensity value of the control group, and FI _dosing_ is the fluorescence intensity value of different dosing groups.

### 2.4 Cell viability assays *in vitro*

After 0.8 × 10^4^ cells per well were spread in 96-well plates and incubated for 24 h, the wells were incubated for 4 h with the ESS extract (0.25, 0.5, 1, and 2 mg/mL) and DdT extract (0.25, 0.5, 1, and 2 mg/mL). After discarding the supernatant, each well was incubated with the MTT solution (0.5 mg/mL) for 4 h, and DMSO was added. Absorbance values were measured at the measurement wavelength of 570 nm and the reference wavelength of 650 nm after shaking for 10 min.

### 2.5 The influence of BODIPY FL histamine of natural products

After 0.8 × 10^4^ cells per well were spread in 96-well plates and incubated for 24 h, the wells were incubated for 4 h with the ESS extract (0.125, 0.25, and 0.5 mg/mL) and DdT extract (0.5, 1, and 2 mg/mL) and DPHD (20 μM). Each well was incubated with 60 μL of the BODIPY FL histamine (0.5 μM) for 30 min, and the fluorescence intensity was detected after six repeated washes with PBS.

### 2.6 Screening the H1R antagonists from natural products by DPHD-anchored bombardment coupled with target cell extraction

Cells were divided into two groups: traditional Chinese medicine (TCM) + DPHD and TCM. First, cells were treated with DdT (2 mg/mL) and ESS (1 mg/mL) for 4 h in the TCM + DPHD group, respectively. Then, cells were washed with PBS six times repeatedly and incubated for 4 h with an excess of the DPHD solution. Then, DdT (2 mg/mL) and ESS (1 mg/mL) were added to the TCM group for 4 h, respectively. Cells were washed six times repeatedly with PBS and incubated for 4 h with serum-free 1640 medium. After washing with PBS, cells were broken by sonication with 80% methanol, and the supernatant was collected. After being evaporated dry, 200 μL methanol was added to reconstitute (12,000 r, 10 min), and the supernatant was taken for high-performance liquid chromatography-quadrupole time-of-flight mass spectrometry (HPLC-Q-TOF-MS) detection.

The Agilent 1260 Infinity HPLC system from Agilent Technologies (Santa Clara, CA, United States) was used for chromatographic separation. An Agilent ZORBAX SB-C18 column (4.6 × 250 mm, 5 μm) was employed. Mass spectrometry data were collected using the Agilent 1260 Q-TOF MS/MS system (Agilent Technologies, Santa Clara, CA, United States), which was equipped with an electrospray ionization (ESI) interface. The operational parameters were set as follows: the ESI source was operated in the positive mode with a nitrogen (N_2_) flow rate of 10.0 L/min for the drying gas. The temperature of the drying gas was maintained at 350°C, and the nebulizer pressure was set to 40 psi. The capillary voltage was adjusted to 4,000 V, while the skimmer voltage was maintained at 65 V. Additionally, the fragment voltage was set to 135 V. The scan range covered a mass range from 50 to 1500 Da.

DdT: the mobile phase consisted of acetonitrile (A) and water (B) with a flow rate of 1.0 mL/min and column temperature 25°C. The detection wavelength used was 254 nm. The elution gradient proceeded as follows: 90%–70% B for 0–5 min; 70%–46% B for 5–30 min; 46%–23% B for 30–50 min; 23%–5% B for 50–51 min; 5%–5% B for 51–61 min.

ESS: the mobile phase consisted of acetonitrile (A) and 0.4% acetic acid–20 mM ammonium acetate–water (B) with a flow rate of 1.0 mL/min and column temperature 30°C. The detection wavelength used was 325 nm. The elution gradient proceeded as follows: 95%–87% B for 0–5 min; 87%–82% B for 5–12 min; 82%–75% B for 12–32 min; 75%–60% B for 32–34 min; 60%–0% B for 34–35 min; and 0%–0% B for 35–45 min.

### 2.7 Evaluation of the effects of potentially active components *in vitro*

#### 2.7.1 Cell viability assays *in vitro*

The control group was given serum-free 1640 medium, and the treatment group was given ephedrine (25, 50, 100, and 200 μM), pseudoephedrine (25, 50, 100, and 200 μM), dictamnine (25, 50, 100, and 200 μM), and limonin (25, 50, 100, and 200 μM). The other operations are the same as in 2.4.

#### 2.7.2 Binding of potentially active components to H1R detected by the BODIPY FL histamine recognition assay

The control group was given serum-free 1640 medium, and the treatment group was given ephedrine (12.5, 25, 50, and 100 μM), pseudoephedrine (25, 50, 100, and 200 μM), dictamnine (25, 50, 100, and 200 μM), and limonin (12.5, 25, 50, and 100 μM). The other procedures are the same as in 2.5.

#### 2.7.3 Intracellular Ca^2+^ fluctuation assay

The cells were inoculated with 0.5 × 10^4^ cells per well in a 96-well fluorescent enzyme plate and incubated for 24 h, which were divided into the histamine group and the administration group, referring to pseudoephedrine (50, 100, and 200 μM) and dictamnine (25, 50, and 100 μM), and incubated for 4 h. Subsequently, 60 μL of Fluo-4 AM (2 μM) was added and cultivated for 40 min and washed with Locke’s buffer six times. The baseline F0 was detected at the excitation wavelength 488 nm and emission wavelength 520 nm, and 100 μL of histamine (50 μM) was added to each well after discarding the supernatant, and it was detected again.

#### 2.7.4 Molecular docking

Molecular docking was carried out by using Schrödinger Maestro chemical simulation software (https://www.schrodinger.com/maestro). The crystal structure of H1R combined with the ligand doxepin (D7V) was downloaded from the RCSB database (https://www.rcsb.org/), PDB code (3RZE), with a resolution of 3.10 Å. Protein structures were optimized using the protein preparation wizard module of the software and given an OPLS-2005 force field for energy minimization. The corresponding grid point files were generated by setting the square boxes of 20 Å _20 Å _20 Å with the original ligand D7V as the active center in the receptor grid generation module. The ligands were downloaded in PubChem (https://pubchem.ncbi.nlm.nih.gov/) in the sdf format and given the OPLS-2005 force field in the LigPrep module of the software, with other parameters unchanged for ligand optimization. The optimized ligand and receptor lattice files were semi-flexible docked using the ligand docking module, maintaining the rigidity of the receptor and varying the ligand within the lattice. Each ligand outputs 10 conformations, and the optimal conformation was selected based on the docking scoring value.

### 2.8 Evaluation of the effects of potentially active components *in vivo*

#### 2.8.1 Animal experiments

A total of 50 female BALB/c mice (18–22 g) were gained from the Huachuang Sino Pharmaceutical Technology Co. (Taizhou, Jiangsu Province, China) with the license number: SCXK (Su) 2020-0009. They were kept in an air-conditioned room at 23°C ± 1°C with a light/dark cycle of 12 h. All animal studies were approved by the Animal Ethics Committee of the China Pharmaceutical University.

The model of allergic rhinitis (AR) mice was constructed (Kim et al., 2016; Van Nguyen et al., 2020; Wei et al., 2021), which was divided into two stages, the basal sensitization phase: ovalbumin (OVA) was used as the sensitizing antigen for modeling, and 200 μL saline containing 50 μg ovalbumin and 2 mg of aluminum hydroxide was intraperitoneally injected. Three sensitizations were performed on days 1, 8, and 15. The challenge phase: 7 days after the end of the basal sensitization phase, once a day for eight times, the ovalbumin solution was instilled into the bilateral nasal cavity of the mice at 20 μL (20 mg/mL) per side.

The 50 female mice were randomly assigned to five groups: the control group, the model group (AR), the low dose of dictamnine group (dictamnine-L, 10 mg/kg), the high dose of dictamnine group (dictamnine-H, 20 mg/kg), and the loratadine group (1.67 mg/kg). The dose group was administered continuously from day 15 to day 29, once daily for 14 days. After 2 weeks, mice were killed to collect nasal tissue and serum for analysis.

#### 2.8.2 Bodyweight and nasal symptom score

Body weight was recorded at days 1 and 29. After the last nasal OVA drop, the number of rubbing and sneezing within 15 min was recorded.

#### 2.8.3 Analysis of serum indicators

Serum cytokine levels of immunoglobulin E (IgE), histamine, interleukin-2 (IL-2), interleukin-4 (IL-4), interleukin-6 (IL-6), and tumor necrosis factor-α (TNF-α) were measured by ELISA using kits from Wuhan Fain Biologicals (Wuhan, China). Each step was strictly determined according to the manufacturer’s instructions.

#### 2.8.4 Histopathology

After the mice were killed, the skin of the maxilla was peeled off, and the nasal bones were stripped. Next, the nasal cavity was cut open to expose the nasal septum, and the bilateral mucosa was peeled off. The nasal mucosa and lungs were immersed in freshly prepared 4% paraformaldehyde and fixed for 48 h. After dehydration, hematoxylin–eosin (HE) and periodic acid-Schiff (PAS) staining were performed. Morphological and structural changes and inflammatory infiltration of the endonasal cavity and lung tissue were observed under the microscope.

### 2.9 Statistical analysis

All data statistics were carried out with GraphPad Prism 8.0.1 software, and experimental data were expressed as mean ± SD. ANOVA was used to compare the data of each group, Student’s test was used to compare the data between two groups, and Dunnett’s test was used to compare the data of three or more groups. *P* < 0.05 indicated that the difference was statistically significant.

## 3 Results

### 3.1 Establishing and optimizing of the BODIPY FL histamine recognition of H1R antagonists

**FIGURE 2:**
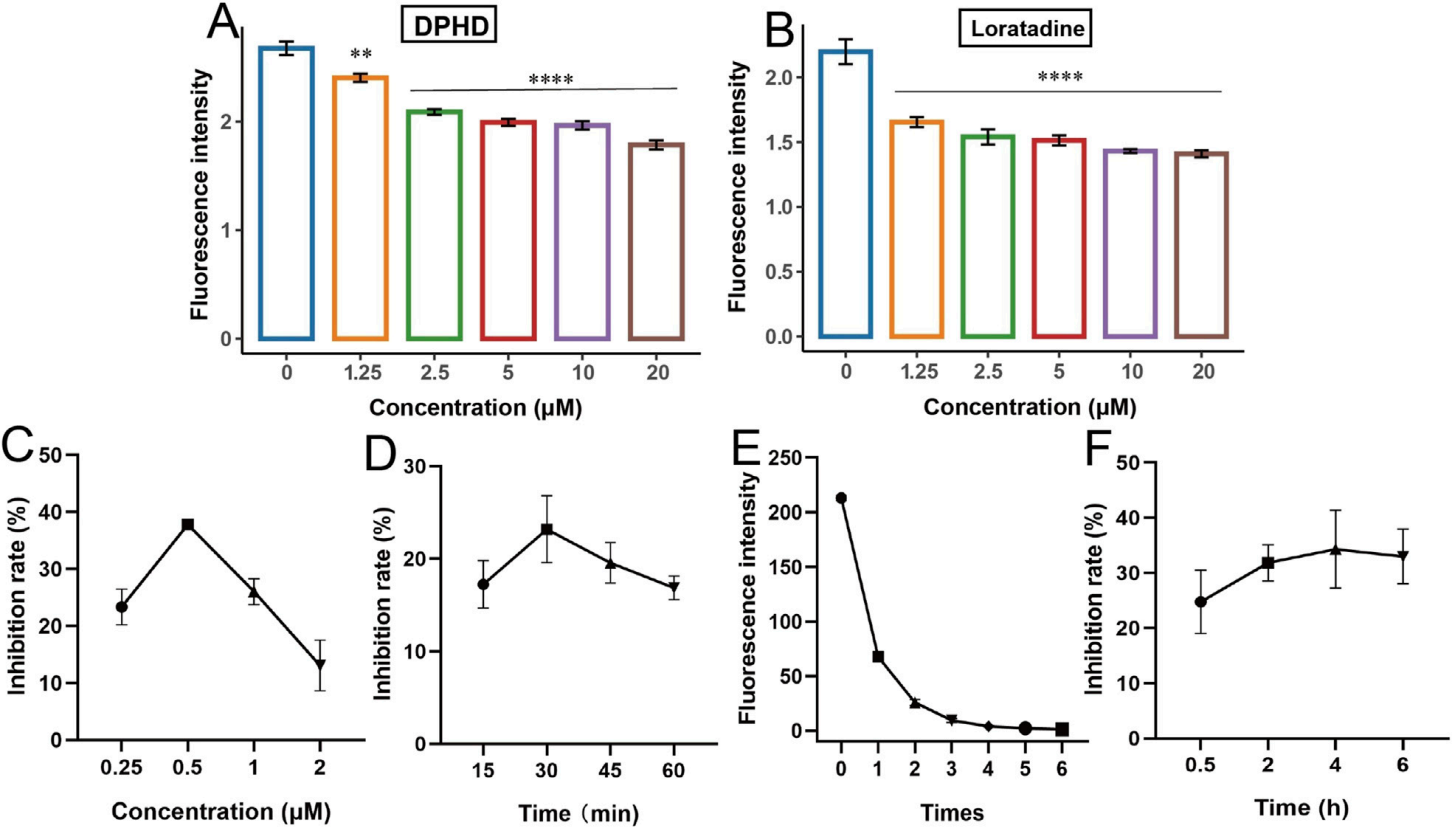
Establishing and optimizing of the BODIPY FL histamine recognition of H1R antagonists. **(A)** DPHD; **(B)** loratadine; **(C)** incubation concentration of BODIPY FL histamine; **(D)** incubation time of BODIPY FL histamine; **(E)** washing times; **(F)** incubation time of diphenhydramine. (**p < 0.01 and ****p < 0.0001 compared to the control group).

As shown in Figures 2A,B, the fluorescence intensity showed a concentration-dependent decrease with increasing concentrations of DPHD and loratadine administered, both of which were significantly different compared with the control group, indicating that both positive drugs inhibited the binding of the BODIPY FL histamine to histamine receptors on HUVECs. This result suggested that H1R antagonists can bind to histamine receptors on HUVECs and the BODIPY FL histamine can be used for the recognition of histamine receptor antagonists.

Next, the detection conditions of the BODIPY FL histamine recognition method were optimized using DPHD as a tool drug. Figure 2C indicated that the maximum inhibition of DPHD was achieved when the concentration of the BODIPY FL histamine was 0.5 μM. Figure 2D showed that the BODIPY FL histamine could achieve maximum inhibition by incubating with the HUVECs for 30 min. Therefore, the subsequent experiments were performed with the BODIPY FL histamine (0.5 μM) incubated with HUVECs for 30 min as the selection condition. Figure 2E shows that the fluorescence intensity value of the sixth washing solution has reached the lowest, indicating that the residual of the BODIPY FL histamine in the supernatant was basically washed out after six washes, and its interference with the detection results can be excluded. As can be seen from Figure 2F, DPHD could achieve the optimal inhibition rate by co-incubating with HUVECs for 4 h. Therefore, 4 h was the optimal incubation time for subsequent natural product identification studies.

### 3.2 The BODIPY FL histamine recognition of the H1R antagonists method applied to identifying TCM extracts

**FIGURE 3:**
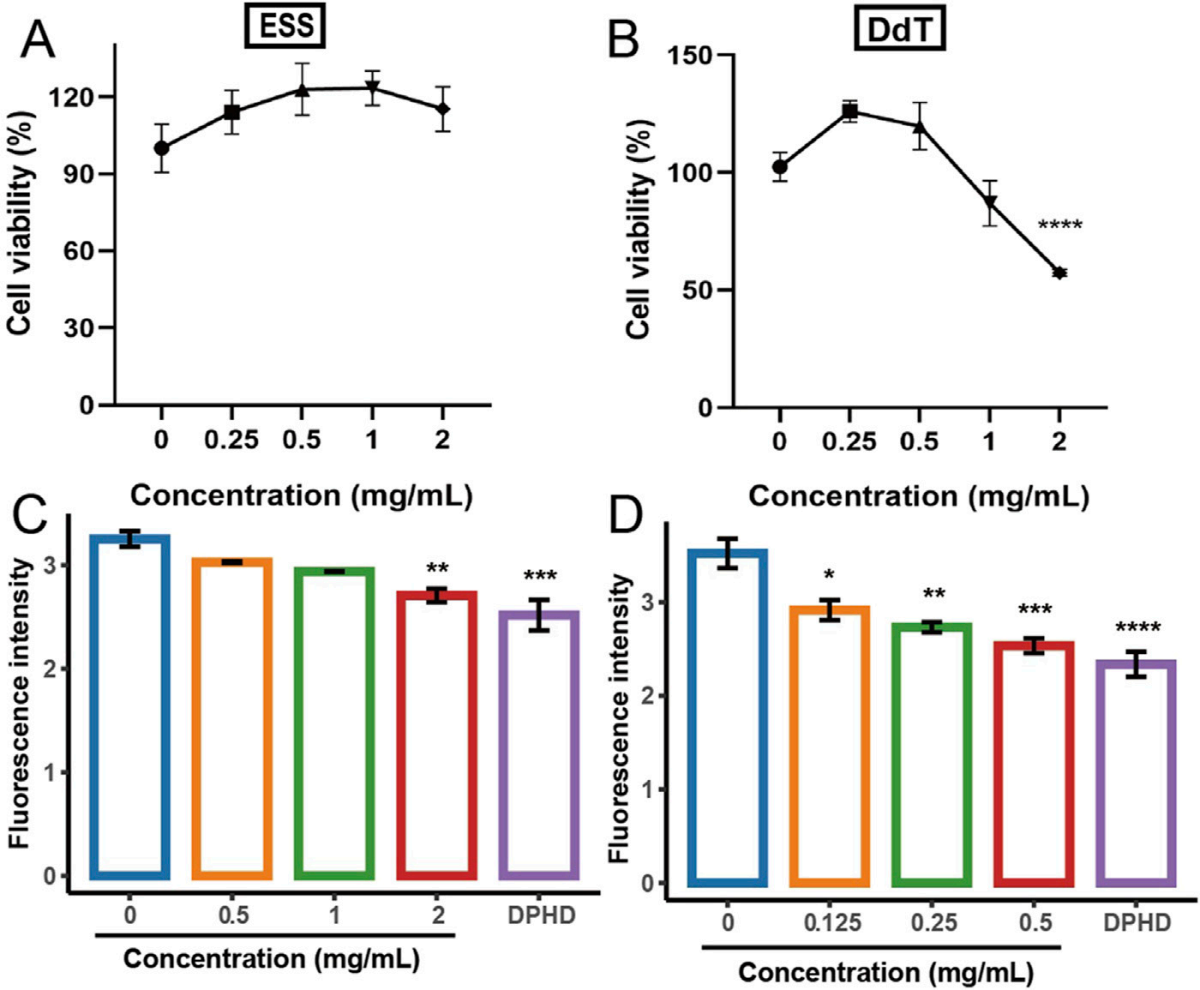
Effect of different extracts on the viability of HUVECs and the BODIPY FL histamine. **(A,C)** ESS; **(B,D)** DdT (*p < 0.05, **p < 0.01, ***p < 0.001, and ****p < 0.0001 compared to the control group).

The effect of DdT and ESS extracts on the viability of HUVECs is shown in Figures 3A,B. Figure 3A shows that the ESS extract had almost no effect on the viability of HUVECs in a certain concentration range. Figure 3B indicates that HUVEC viability was significantly decreased by DdT extracts at a concentration of 2 mg/mL compared to that in the control group.

The optimized method was applied for the identification of two natural products. Figure 3C shows the inhibition of BODIPY FL histamine binding to histamine receptors by the ESS, and compared with the control group, the inhibition of DPHD (*p* < 0.001) and ESS (2 mg/mL, *p* < 0.01) was significant. The fluorescence intensity gradually decreased with the increase in the concentration of the total extract of ESS. The results suggested that the ESS may contain potential active components that can antagonize H1R activity, which was closely related to its anti-allergic effect. The results of the inhibition of BODIPY FL histamine by total extracts of DdT are shown in Figure 3D. With the increase in the concentration of DdT, the fluorescence intensity showed a concentration-dependent decrease, all of which were significantly different than that in the control group. The data indicated that the components in DdT bound to the histamine receptor of HUVECs, subsequently inhibiting the binding of fluorescent histamine. The results suggested that DdT may contain active components that can antagonize H1R, which is closely related to its mechanism of treating allergic diseases.

### 3.3 Screening and identification of the H1R antagonists from DdT and ESS by DPHD-anchored bombardment coupled with target cell extraction

**FIGURE 4:**
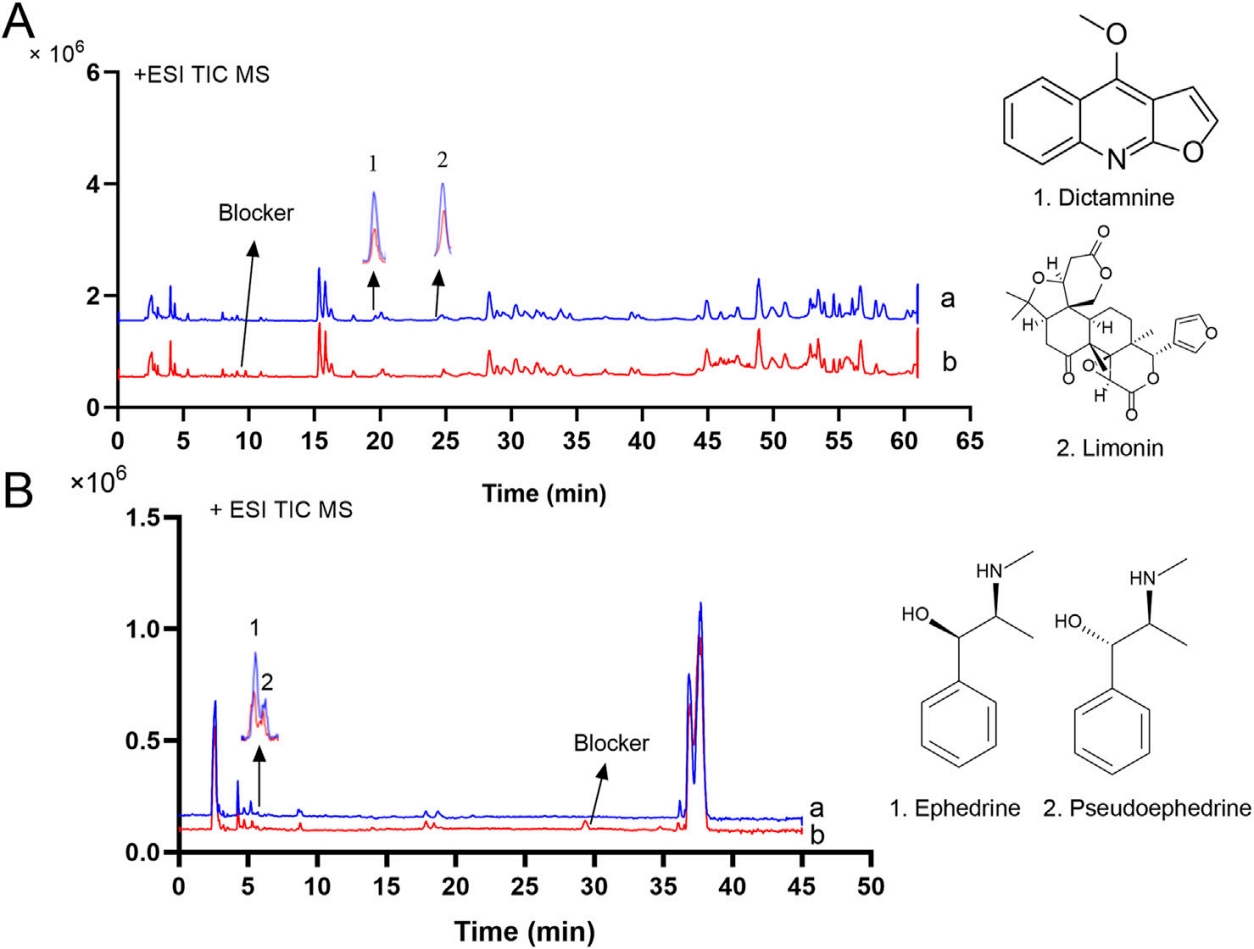
MS chromatogram and structural formula of potential active components of the eluent of HUVECs binding with DdT [**(A)**: a: DdT; b: DdT+DPHD)] and ESS [**(B)**: a: ESS; b: ESS+DPHD)].

Mass spectrometry analysis was conducted in the positive ion mode on cellular samples extracted from various groups. The total ion flow diagrams of DdT are presented in Figure 4A, with group a (DdT) and group b (DdT + DPHD). The total ion flow diagrams of ESS are presented in Figure 4B, with group a (ESS) and group b (ESS + DPHD). DPHD-anchored bombardment of H1R binding sites on HUVEC membranes results in a significant reduction of the components in herbal extracts that are bound to these H1R sites. By extracting the ion flow, a significant difference in the content of the two components was observed between the two groups. This suggested that the DPHD-anchored bombardment of the H1R-binding site led to a reduction in the content of H1R-binding components in the DdT and ESS. Comparing the reference information and the PubChem database, these components were identified as dictamnine (Chang et al., 2021), limonin (Liu et al., 2017), ephedrine, and pseudoephedrine (Sun et al., 2016; Guo et al., 2018; Song et al., 2024). The results of mass spectrometry identification and structural information are shown in Supplementary Table S1. It was stated that dictamnine, limonin, ephedrine, and pseudoephedrine may be the active components with the potential to antagonize histamine H1R.

### 3.4 Evaluation of the effects of potentially active components *in vitro*

#### 3.4.1 The effect of potential active ingredients on HUVECs

**FIGURE 5:**
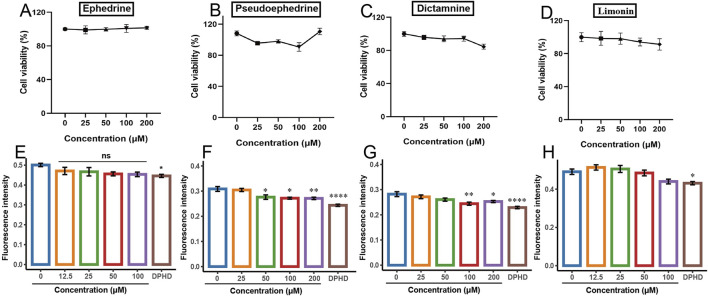
Effect of different potentially active components on the viability of HUVECs and the BODIPY FL histamine. **(A,E)** ephedrine; **(B,F)** pseudoephedrine; **(C,G)** dictamnine; **(D,H)** limonin. (*p < 0.05, **p < 0.01, ***p < 0.001, and ****p < 0.0001 compared to the control group).

Figures 5A,B,D illustrate the impact of varying concentrations of ephedrine, pseudoephedrine, and limonin on cell viability, respectively. The results indicated that within the concentration range of 0–200 μM, these three monomers exhibited minimal toxicity to the cells, with cell survival rates being approximating 100%. Conversely, Figure 5C demonstrates the effect of various concentrations of dictamnine on the viability of HUVECs. Cells treated with concentrations of 50, 100, and 200 μM exhibited a notable decrease in viability compared to the control group, albeit with the cell viability remaining above 80%.

#### 3.4.2 The effect of potentially active ingredients on the BODIPY FL histamine

The effects of the four monomers on the binding of the BODIPY FL histamine to H1R are presented in Figure 5. In contrast to the control group, DPHD significantly decreased the fluorescence intensity. Figures 5E–H depict the impact of ephedrine and limonin on the fluorescence intensity of BODIPY FL histamine bound to H1R. The results indicated a trend toward decreased fluorescence intensity with increasing concentrations in the treated groups, but this difference was not statistically significant compared to the control group. Figure 5F illustrates that as the concentration of pseudoephedrine increased, the binding of BODIPY FL histamine to H1R decreased in a concentration-dependent manner. Figure 5G demonstrates that dictamnine (100 μM) significantly reduced the binding of BODIPY FL histamine to H1R compared to that in the control group (*p* < 0.01). These findings suggested that dictamnine and pseudoephedrine can bind to H1R, thereby reducing the binding of BODIPY FL histamine. Consequently, dictamnine and pseudoephedrine may serve as potential H1R blockers in the total extracts of DdT and ESS, respectively.

#### 3.4.3 The effect of potentially active ingredients on the intracellular Ca^2+^ fluctuation assay

Numerous studies showed that the histamine-induced increase of the Ca^2+^ concentration in HUVECs is closely related to H1R, which was attributed to the fact that binding of histamine to H1R on HUVECs leads to the activation of calcium channels (Bosma et al., 2016; Hou et al., 2019). Research indicated that siRNA-mediated knockdown of H1R in HUVECs attenuated the histamine-induced elevation of intracellular calcium concentration (Cao et al., 2020). Additionally, H1R antagonists counteracted the binding of histamine to H1R on HUVECs, leading to a reduction in the histamine-induced increase in intracellular calcium concentration (Burghi et al., 2021).

**FIGURE 6:**
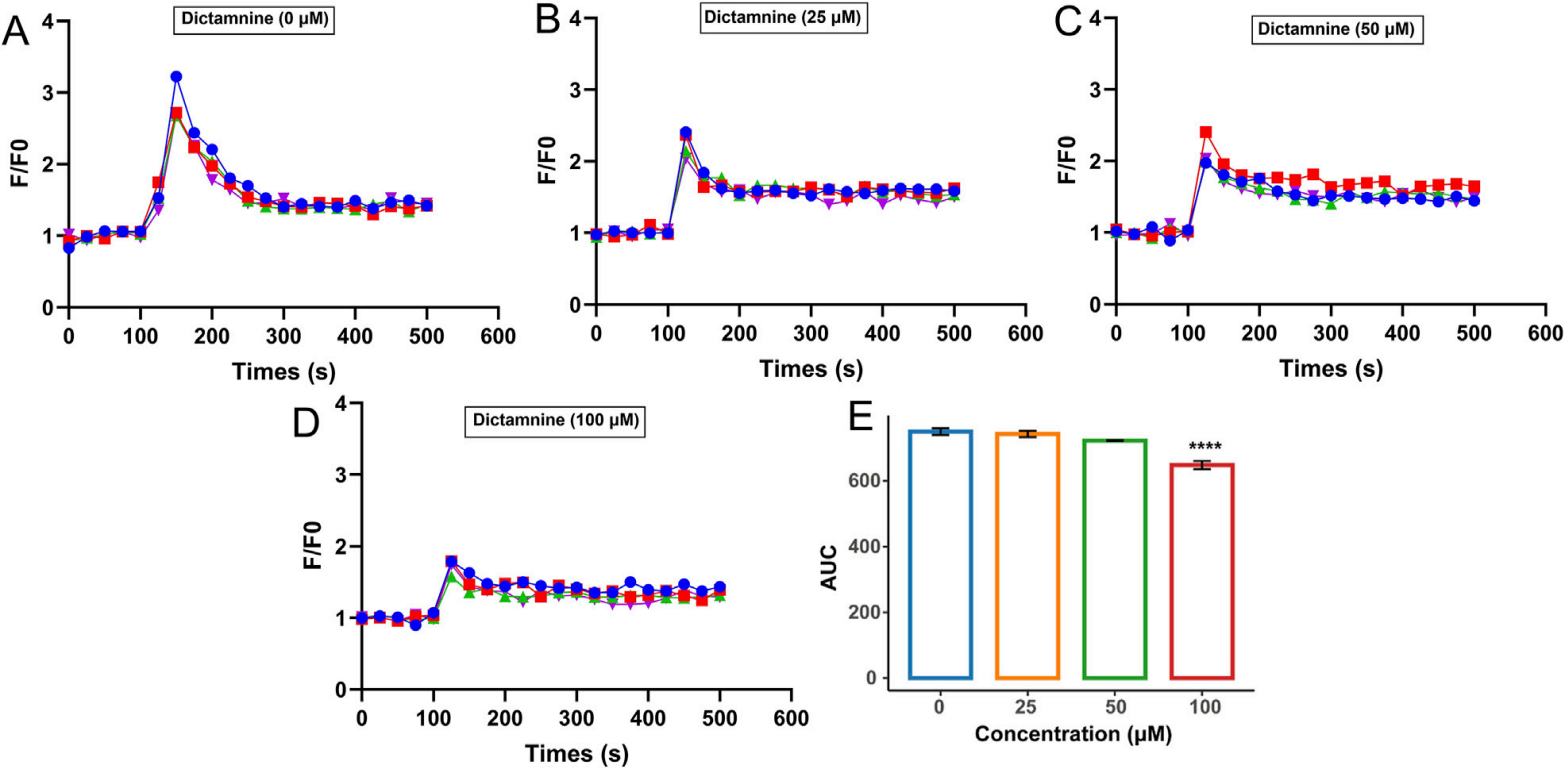
Inhibition effect of dictamnine on intracellular Ca^2+^ increase induced by histamine. Concentration of dictamnine: **(A)** 0; **(B)** 25 μM; **(C)** 50 μM; **(D)** 100 μM. **(E)** AUC statistic of the above groups (****p < 0.0001, compared with the histamine group-dictamnine 0 μM).

The potential active ingredients were further validated using a previous method of establishing fluorescence detection Ca^2+^ signaling (Hu et al., 2021). The effects of dictamnine on histamine-induced increases in intracellular Ca^2+^ concentration in HUVECs are illustrated in Figures 6A–E. Figure 6A depicts the change in intracellular Ca^2+^ concentration induced by 50 μM histamine acting on the H1R in HUVECs. Figures 6B–D show the changes in intracellular Ca^2+^ concentration induced by the stimulation of the cells with histamine after the incubation of dictamnine (25, 50, and 100 μM), respectively. These results indicated that the histamine-induced increase in intracellular Ca^2+^ concentration decreased with increasing dictamnine incubation concentration. The areas under the curves for Figures 6A–D were calculated and presented in Figure 6E. It was clearly observed that dictamnine concentration-dependently reduced the intracellular Ca^2+^ concentration, with the inhibitory effect of dictamnine (100 μM) being significantly different (*p* < 0.0001). These findings further confirmed that dictamnine may serve as an effective component in DdT for blocking histamine binding to H1R.

**FIGURE 7:**
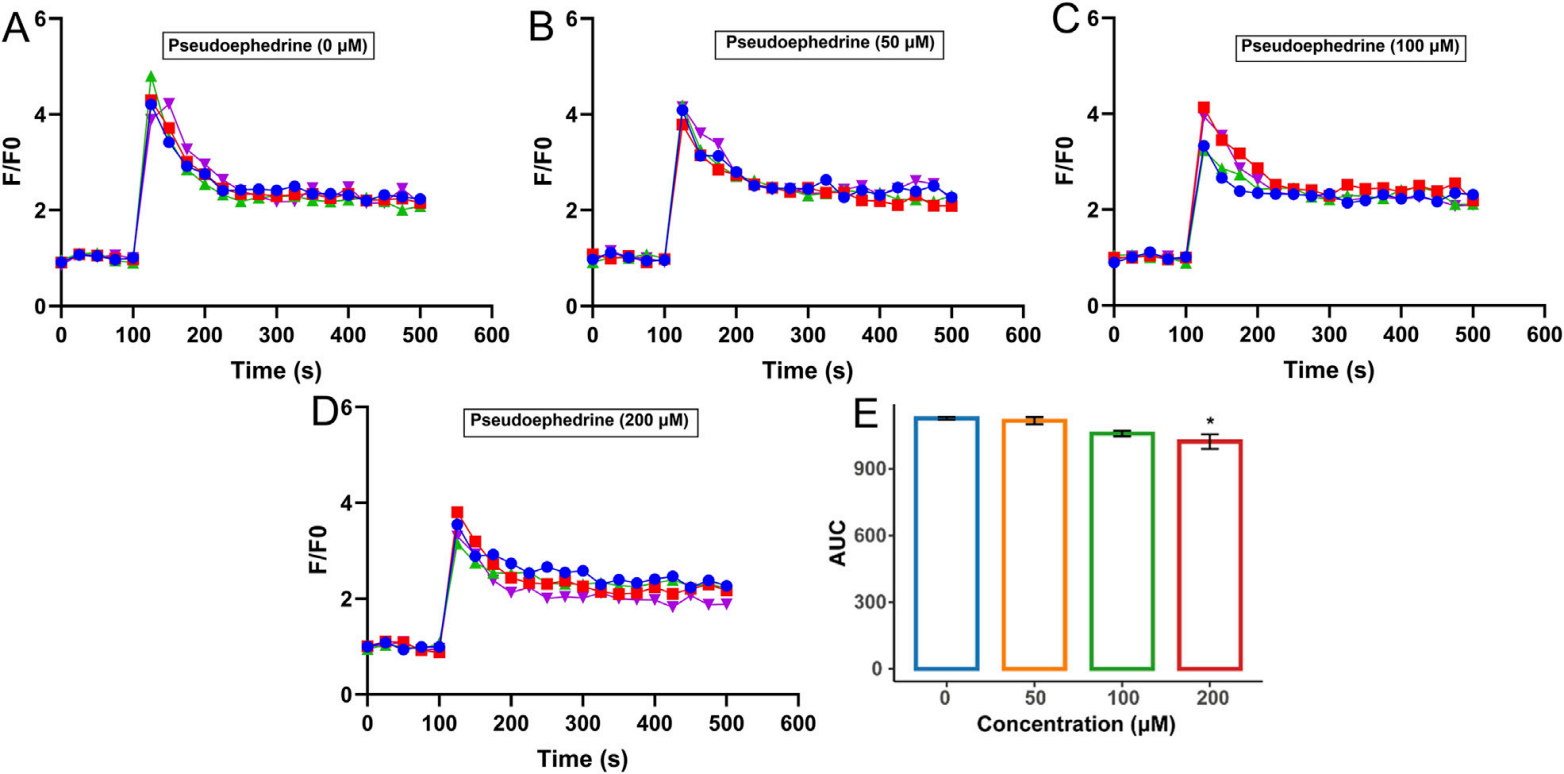
Inhibition effect of pseudoephedrine on intracellular Ca^2+^ increase induced by histamine. Concentration of pseudoephedrine: **(A)** 0; **(B)** 50 μM; **(C)** 100 μM; **(D)** 200 μM, **(E)** AUC statistic of the above groups (*p < 0.05, compared with the histamine group-pseudoephedrine 0 μM).

In the same way, the effects of pseudoephedrine on histamine-induced increases in intracellular Ca^2+^ concentration in HUVECs are illustrated in Figures 7A–E. The areas under the curves for Figures 7A–D were calculated and are presented in Figure 7E. It was clearly observed that pseudoephedrine concentration-dependently reduced the intracellular Ca^2+^ concentration, with the inhibitory effect of pseudoephedrine (200 μM) being significantly different (*p* < 0.05). These findings further confirmed that pseudoephedrine may serve as an effective component in ESS for blocking histamine binding to H1R.

#### 3.4.4 Docking results

**FIGURE 8:**
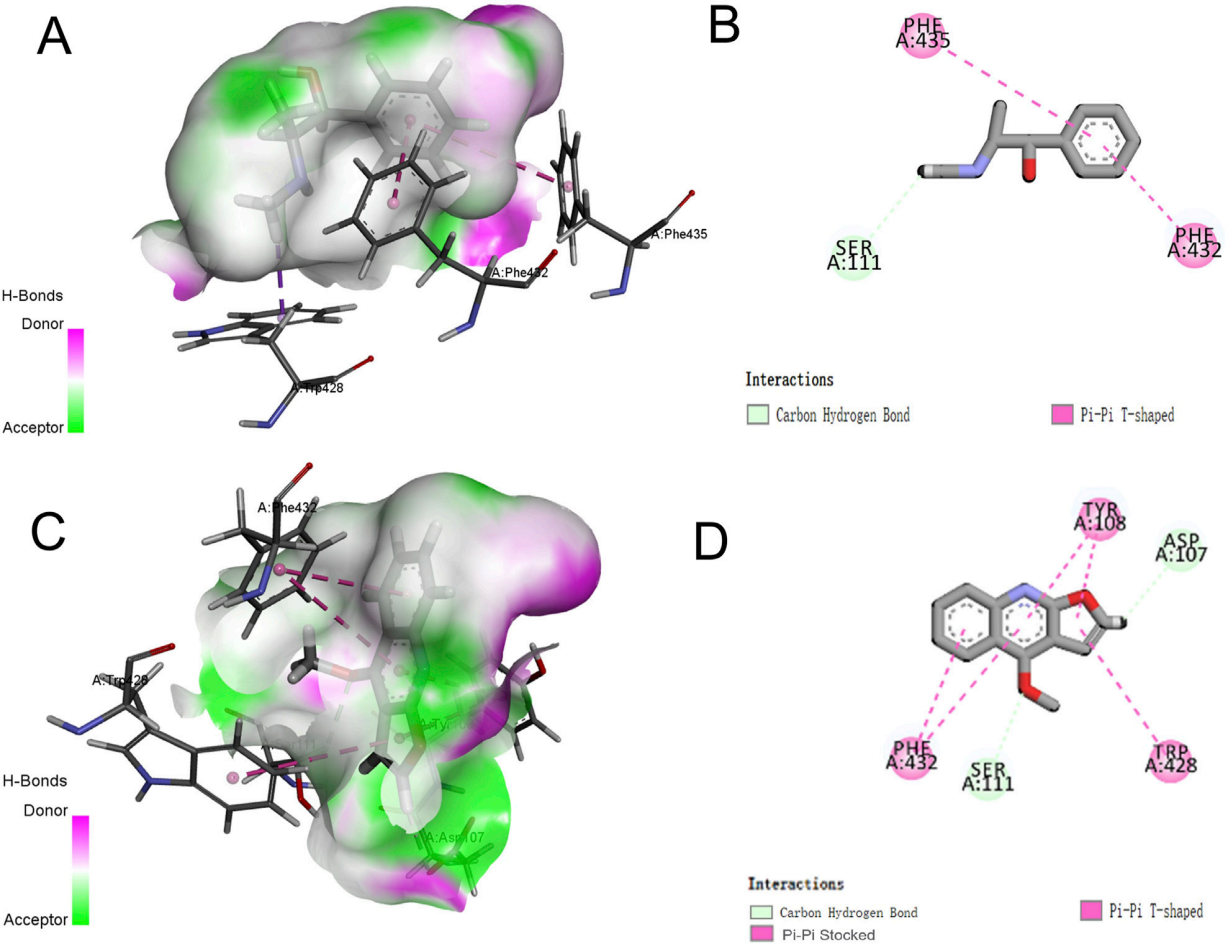
Three-dimensional and two-dimensional docking models of components with H1R generated by Discovery Studio Visualizer. **(A,B)** Pseudoephedrine; **(C,D)** dictamnine.

The docking results of pseudoephedrine and dictamnine with H1R are presented in Figures 8A,B, depicting the 3D and 2D diagrams of the docking of pseudoephedrine with H1R, respectively. Pseudoephedrine bound to the amino acid residue SER111 of H1R through a carbon–hydrogen bond, while residues PHE435 and PHE432 interacted with H1R via Pi–Pi T-shaped bonds, whose docking score was −6.975. Previous studies have indicated that amino acid residues Asp107, Tyr108, Trp428, Tyr431, Phe432, and Phe435 within the transmembrane domain of H1R contribute to the formation of lipophilic pockets in the binding cavity of histamine antagonists (Söldner et al., 2018). Supplementary Table S2 summarizes the amino acid residues involved in binding both compounds to H1R, along with their respective docking scores. Figures 8C,D illustrate the 3D and 2D diagrams of the interaction between dictamnine and H1R. As shown in Figure 8D, dictamnine formed carbon–hydrogen bonds with ASP107 and SER111 and Pi–Pi bonds with TYR108, TRP428, and PHE432, whose docking score was −7.460. The results suggested that pseudoephedrine and dictamnine may serve as potential H1R antagonists. Notably, dictamnine exhibited a higher scoring value compared to pseudoephedrine, indicating a stronger binding affinity with H1R than pseudoephedrine.

### 3.5 Evaluation of the effects of potentially active components *in vivo*

#### 3.5.1 Dictamnine improves the nasal symptom score and serum indicators

**FIGURE 9:**
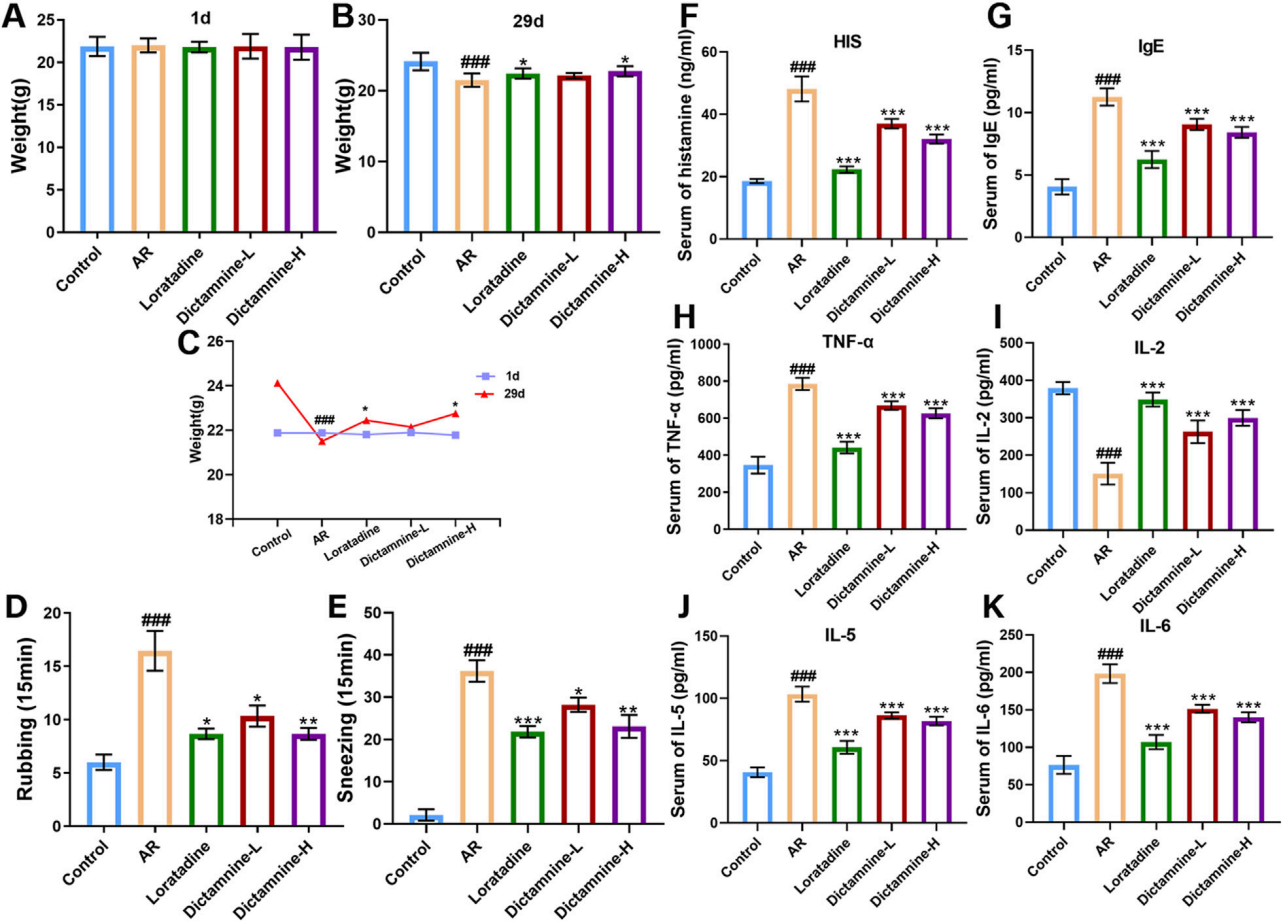
Effects of dictamnine on body weight, behavior, and serum cytokine levels of AR mice. **(A)** Initial weight; **(B)** final weight; **(C)** weight curve, **(D)** rubbing; **(E)** sneezing, **(F)** histamine (HIS); **(G)** IgE; **(H)** TNF-α; **(I)** IL-2; **(J)** IL-5; **(K)** IL-6 (###p < 0.001 compared to the control group; *p < 0.05, **p < 0.01, and ***p < 0.001 compared to the model group).

The effects of dictamnine on body weight, behavior, and serum cytokine levels of AR mice are presented in Figure 9. Compared with those in the control group, the body weight of AR mice increased slowly (Figures 9A–C). Previous studies have reported that AR mice may exhibit phenomena such as hair loss, reduced food intake, and slow weight gain (Chen F. et al., 2020; Zhou et al., 2024). Dictamnine-H could significantly ameliorate weight loss in AR mice (*p* < 0.05). As shown in Figures 9D,E, dictamnine could dose-dependently reduce the rubbing and sneezing times of AR mice. Compared with the levels of histamine, IgE, IL-5, IL-6, and TNF-α in the AR group, there was a dose-dependent improvement caused by dictamnine (*p* < 0.001, Figures 9F–K). Compared with the levels of IL-2 in the model group, dictamnine could dose-dependently increase the levels (*p* < 0.001, Figure 8I).

#### 3.5.2 Dictamnine improves the pathological state of nasal mucosa and lung

**FIGURE 10:**
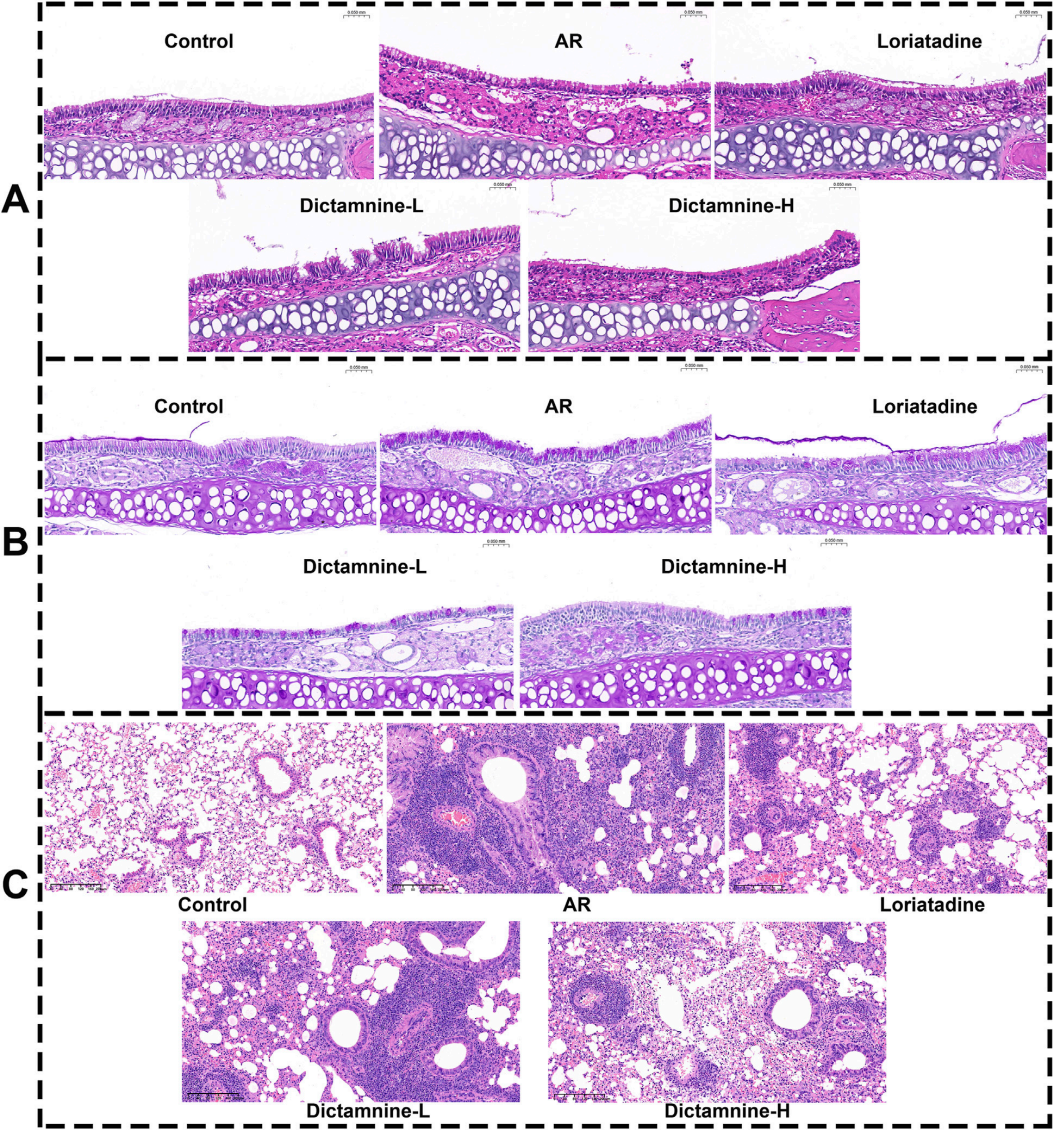
Effect of dictamnine on histological changes in the nasal mucosa and lung of AR mice. **(A)** HE staining (nasal mucosa, 200x); **(B)** PAS staining (nasal mucosa, 200x); **(C)** HE (lung, 400x).

The results of HE and PAS staining showed that the nasal mucosal epithelial cells in the control group mice were neatly arranged, the ciliated structure was intact without congestion, and the columnar cell layer was intact and undamaged. Conversely, the nasal mucosal epithelial cells in the model group were disarrayed, showing congestion and edema. The basement membrane was detached. There was a marked increase in goblet cell infiltration, numerous inflammatory cells, and extensive detachment of cilia. The phenomena of congestion and edema of the nasal mucosa, infiltration of inflammatory cells, goblet cell proliferation, detachment of the cilia, and proliferation of the glands were improved in dictamnine-L and dictamnine-H groups (Figures 10A,B).

The results of HE staining showed that the lung tissue of the control group mice showed normal morphology, with clear alveolar, bronchial and interstitial structures, no congestion and edema, and no inflammatory cell infiltration. The lung tissue of AR mice exhibited significant enlargement, accompanied by severe hemorrhage and extensive infiltration of inflammatory cells surrounding the bronchioles and alveoli. No obvious hemorrhage and edema were seen in the alveoli of the lung tissue of dictamnine-L and dictamnine-H groups, and the inflammatory cell infiltration was significantly decreased (Figure 10C).

## 4 Discussion

Histamine action on H1R on endothelial cells leads to vasodilation and increased permeability, triggering common allergic diseases such as edema, AR, and asthma (Branco et al., 2018; Gao et al., 2022). HUVECs have relatively high levels of H1R receptor expression (Cao et al., 2020; He et al., 2020). Numerous studies have shown that BODIPY FL histamine is an effective tool for studying cell surface receptors (Mirzahosseini et al., 2015; Mocking et al., 2018; Soave et al., 2021). In this study, the BODIPY FL histamine recognition method of H1R antagonists was constructed with HUVECs with the help of H1R antagonist DPHD (DPHD).

All current H1 receptor antagonists are nitrogen-containing compounds, which can be categorized into structural classes such as piperidines, ethanolamines, and ethylenediamines. Consequently, it was inferred that alkaloid components constitute the primary compounds that antagonize H1R. Therefore, the alkaloid-rich DdT and ESS were chosen as research objects. DdT, a traditional Chinese medicine commonly prescribed for dermatological disorders, exhibited anti-inflammatory and anti-allergic effects (Lv et al., 2015; Qin et al., 2021). Park et al. demonstrated that DdT extracts can improve skin barrier function and symptoms of atopic dermatitis in mice (Park et al., 2024). Yang et al. demonstrated that DdT extracts could inhibit oxazolone-induced allergic dermatitis (Kim et al., 2019). ESS possessed asthma-calming and anti-metamorphic properties and was a widely utilized traditional Chinese medicine for clinical anti-allergic treatments (Zheng et al., 2023). ESS may act as an anti-allergic agent by inhibiting the release of allergic mediators (Tang et al., 2023), and it may also exert a calming effect by vasoconstriction (Miao et al., 2020). There are no studies that have reported on whether the anti-allergic actions of these two herbs are associated with antagonism of H1R binding. Our results suggested that the ESS and DdT may contain potential active components that can antagonize H1R, which was closely related to their anti-allergic effect. In order to accurately screen the components of natural products bound to H1R on HUVECs by target cell extraction, the concept of DPHD “anchoring bombardment” was introduced based on the specific binding of H1R by DPHD. The results of mass spectrometry show that dictamnine, limonin, ephedrine, and pseudoephedrine may be the active components with the potential to antagonize histamine H1R.

Next, the effects of the four monomers on the binding of the BODIPY FL histamine to H1R were analyzed, which showed that dictamnine and pseudoephedrine can bind to H1R, thereby reducing the binding of BODIPY FL histamine. Dictamnine was the primary active component of DdT, which could regulate the degranulation of mast cells in atopic dermatitis mice (Lin et al., 2021) and ameliorate inflammation in an oxazolone-induced dermatitis mouse model (Huang et al., 2024). Liu et al. discovered that dictamnine exhibited the ability to ameliorate hypersensitivity through its binding to MrgX2 receptors on mast cells (Liu et al., 2021), and it can ameliorate the AR via suppression of the LYN kinase-mediated molecular signaling pathway during mast cell activation (Liu et al., 2023). Pseudoephedrine was the main active ingredient of ESS, which could improve allergic rhinitis (Tang et al., 2015). Some studies showed that pseudoephedrine can significantly improve acute rhinitis in combination with anti-allergic drugs (Grubbe et al., 2009) and alleviate atopic dermatitis-like inflammatory responses (Chen X. et al., 2020). Pseudoephedrine has a vasoconstrictive effect and regulates blood pressure (Zheng et al., 2023). To date, few studies have investigated whether the anti-allergic effect of dictamnine and pseudoephedrine was mediated by the H1R. Our results suggested that dictamnine and pseudoephedrine could reduce the intracellular Ca^2+^ concentration histamine-induced increase by using the previous method of establishing fluorescence detection Ca^2+^ signaling. In addition, the docking results also suggested that pseudoephedrine and dictamnine may serve as potential H1R antagonists.

AR is one of the most common clinical allergic diseases. H1R antagonists are currently the first-line drugs for the treatment of AR (Wang et al., 2021). Therefore, the efficacy of dictamnine was further observed at the animal level (AR mice). The results showed that dictamnine could dose-dependently reduce the rubbing and sneezing times and serum indicators (histamine, IgE, IL-5, IL-6, IL-2, and TNF-α) of AR mice. In addition, it could improve the pathological state of the nasal mucosa and lung. Finally, our results showed that dictamnine (validated *in vitro* and *in vivo*) and pseudoephedrine (validated *in vitro*) may serve as potential H1R antagonists.

## 5 Conclusion

In this study, the BODIPY FL histamine was employed to develop a rapid identification method for selecting H1R antagonists. Initially, the method was optimized using DPHD as a model compound, revealing that both ESS and DdT extracts exhibited robust blocking effects on the binding of fluorescent histamine to the H1R in HUVECs. Subsequently, the potential H1R active ingredients were screened in ESS and DdT, specifically ephedrine and pseudoephedrine in ESS and dictamnine and limonin in DdT, utilizing the DPHD-anchored bombardment coupled with target cell extraction. These four compounds were further validated for their pharmacodynamic effects through BODIPY FL histamine recognition, fluorescence detection Ca^2+^ kinetic method, and molecular docking. The results of the BODIPY FL histamine recognition assay demonstrated that pseudoephedrine (50 μM) and dictamnine (100 μM) significantly inhibited the binding of fluorescent histamine to H1R. Concurrently, pseudoephedrine (200 μM) and dictamnine (100 μM) markedly reduced the intracellular Ca^2+^ concentration. Molecular docking results indicated that the amino acid residues bound to H1R by these two compounds contributed to the formation of the active pocket of the histamine antagonist site, with dictamnine exhibiting stronger binding affinity than pseudoephedrine.

Finally, utilizing AR mice as the subject of investigation, dictamnine was proven to significantly alleviate rubbing and sneezing behaviors and dose-dependently decrease histamine and IgE, upregulate IL-2 levels, and downregulate TNF-α, IL-6, and IL-5 levels in serum. Dictamnine effectively improved the pathological state of the nasal mucosa and lung. The ameliorative effect of dictamnine on AR was confirmed through behavioral assessments, serum pharmacodynamic indices, and histological sections. This study showed that dictamnine (validated *in vitro* and *in vivo*) and pseudoephedrine (validated *in vitro*) may serve as potential H1R antagonists. The study provides valuable insights for the subsequent development of antihistamines and novel strategies for screening active ingredients targeting G protein-coupled receptors.

## Data Availability

The raw data supporting the conclusions of this article will be made available by the authors, without undue reservation.
