# Supplementary material for: Screening and identification of the H1R antagonists from natural products by BODIPY FL histamine recognition and DPHD-anchored bombardment coupled with target cell extraction
Source: Front Pharmacol. 2025 May 30;16:1601384. doi: 10.3389/fphar.2025.1601384 (PMC12163627; doi:10.3389/fphar.2025.1601384)
Supplement: Supplementary file 1 [file Table1.docx]

**Supplementary Materials**

**Table S1.** HPLC–Q–TOF–MS/MS characterization of components combination with H1R from DdT and ESS.

**Table S2.** The results of molecular docking.

Table S1 HPLC–Q–TOF–MS/MS characterization of components combination with H1R from DdT and ESS.

| Groups | No. | t_R_ (min) | m/z | Fragment ions | identification | Formula | Structure | error |
| --- | --- | --- | --- | --- | --- | --- | --- | --- |
| DdT | 1 | 19.671 | 200.0707 | 185.0470  129.0568 | Dictamnine | C_12_H_9_NO_2_ |  | -0.48 |
|  | 2 | 24.470 | 471.2013 | 425.1939  161.0599 | Limonin | C_26_H_30_O_8_ |  | -0.97 |
| DdT +DPHD | 1 | 19.668 | 200.0709 | 185.0461  129.0581 | Dictamnine | C_12_H_9_NO_2_ |  | -1.48 |
|  | 2 | 24.496 | 471.2023 | 425.1944  161.0550 | Limonin | C_26_H_30_O_8_ |  | -2.03 |
| ESS | 1 | 6.690 | 166.1227 | 148.1119  133.0894  117.0903 | Ephedrine | C_10_H_15_NO |  | -0.36 |
|  | 2 | 7.008 | 166.1224 | 148.1121  133.0894 | Pseudoephedrine | C_10_H_15_NO |  | 1.46 |
| ESS+DPHD | 1 | 6.659 | 166.1224 | 148.1110  133.0876  117.0702 | Ephedrine | C_10_H_15_NO |  | 1.46 |
|  | 2 | 7.064 | 166.1226 | 148.1115  117.9313 | Pseudoephedrine | C_10_H_15_NO |  | 0.25 |

Table 2 The results of molecular docking

| Compounds | Docking scores | Amino acid residue |
| --- | --- | --- |
| Pseudoephedrine | -6.975 | PHE435, PHE432, SER111 |
| Dictamnine | -7.460 | TYR108, ASP107, TRP428, SER111, PHE432 |
